# Evaluating the scientific reliability of ChatGPT as a source of information on asthma

**DOI:** 10.1016/j.jacig.2024.100330

**Published:** 2024-08-28

**Authors:** Simon Høj, Simon Francis Thomsen, Charlotte Suppli Ulrik, Hanieh Meteran, Torben Sigsgaard, Howraman Meteran

**Affiliations:** a Department of Dermatology, Venereology, and Wound Healing Centre, Copenhagen University Hospital–Bispebjerg, Bispebjerg, Denmark; b Department of Biomedical Sciences, University of Copenhagen, Copenhagen, Denmark; c Department of Public Health, Environment, Occupation, and Health, Aarhus University, Aarhus, Denmark; d Department of Respiratory Medicine, Copenhagen University Hospital–Hvidovre, Hvidovre, Denmark; e Department of Clinical Medicine, University of Copenhagen, Copenhagen, Denmark; f Department of Internal Medicine, Section of Endocrinology, Copenhagen University Hospital–Hvidovre, Hvidovre, Denmark; g Department of Respiratory Medicine, Zealand University Hospital Roskilde–Næstved, Næstved, Denmark

**Keywords:** Asthma, artificial intelligence, patient education, AI, ChatGPT

## Abstract

**Background:**

This study assessed the reliability of ChatGPT as a source of information on asthma, given the increasing use of artificial intelligence–driven models for medical information. Prior concerns about misinformation on atopic diseases in various digital platforms underline the importance of this evaluation.

**Objective:**

We aimed to evaluate the scientific reliability of ChatGPT as a source of information on asthma.

**Methods:**

The study involved analyzing ChatGPT’s responses to 26 asthma-related questions, each followed by a follow-up question. These encompassed definition/risk factors, diagnosis, treatment, lifestyle factors, and specific clinical inquiries. Medical professionals specialized in allergic and respiratory diseases independently assessed the responses using a 1-to-5 accuracy scale.

**Results:**

Approximately 81% of the responses scored 4 or higher, suggesting a generally high accuracy level. However, 5 responses scored >3, indicating minor potentially harmful inaccuracies. The overall median score was 4. Fleiss multirater kappa value showed moderate agreement among raters.

**Conclusion:**

ChatGPT generally provides reliable asthma-related information, but its limitations, such as lack of depth in certain responses and inability to cite sources or update in real time, were noted. It shows promise as an educational tool, but it should not be a substitute for professional medical advice. Future studies should explore its applicability for different user demographics and compare it with newer artificial intelligence models.

## Introduction

Access to accurate and reliable information is crucial for effective asthma management, empowering patients with the knowledge to make informed decisions about their health. The rapid advancement of artificial intelligence (AI) has given rise to AI-driven generative models, such as ChatGPT, as potential sources of medical information.^1^ However, there have been reports casting doubt on their credibility, and previous studies have identified a significant presence of misinformation about atopic diseases on various platforms.^2^, ^3^, ^4^, ^5^ This study evaluated the scientific reliability of ChatGPT as a source of information on asthma, focusing on its accuracy, reliability, and limitations.

To assess ChatGPT’s accuracy, we compiled 25 questions and 1 follow-up question about asthma covering the following aspects: definition/risk factors, diagnosis, treatment, lifestyle factors, and specific clinical questions (Table I). The questions were presented in English as specified in Table I, and both questions and responses were recorded. A panel of 5 medical professionals with specialized knowledge in allergic and respiratory diseases independently evaluated the accuracy and quality of ChatGPT’s answers using a standardized scoring system. The scoring ranged from 1 to 5, representing different levels of accuracy: 1, very poor/unacceptable inaccuracies; 2, poor/minor potentially harmful inaccuracies; 3, moderate/potentially misinterpretable inaccuracies; 4, good/only minor nonharmful inaccuracies; and 5, very good/no inaccuracies. This method has previously been used to examine the quality of answers given by ChatGPT.^6^^,^^7^

**Table I:** Questionnaire summary

Category	Questions
General summary	1. What is asthma?
Symptoms	2. What are the most characteristic symptoms of asthma?
Risk factors	3. What are the risk factors for developing asthma?
	4. What are the triggers of asthma?
Diagnose	5. How do you diagnose asthma?
	6. Is there more than one type of asthma?
	7. What are the most important biomarkers in asthma? Please list them after relevance.
	8. How do you assess the severity of asthma?
	9. What is the most sensitive test for asthma?
	10. Are you sure about that?
Treatment	11. What is the best treatment for asthma listed after relevance?
	12. What is the cornerstone of asthma treatment?
	13. What are the nonpharmacologic treatment options for asthma?
	14. What is the level of evidence on alternative therapies for asthma?
	15. If an adult man only has a few symptoms per month, can he then receive SABAs whenever he has symptoms?
	16. What if a child aged 7 only has symptoms once or twice per month? Is it then appropriate to only receive SABAs?
Complications	17. What are the complications of asthma?
Prognosis	18. What is the prognosis for asthma?
Specific questions	19. Can you treat asthma with means other than medication?
	20. Can you treat asthma with diet?
	21. Can you treat asthma with exercise?
	22. If I have been smoking for 20 years and I have an obstructive lung function. Do I then have COPD or asthma?
	23. I get dyspnea and dizzy when I do sport. Can those symptoms be a sign of asthma?
	24. Are sleep disturbances, tiredness, and loss of concentration symptoms of asthma?
	25. I have an acute worsening of my asthma symptoms. What should I do?
	26. Should I stop doing sport if it triggers my asthma?

*COPD,* Chronic obstructive pulmonary disease; *SABA,* short-acting β-agonist.

## Results and discussion

The responses generated by ChatGPT displayed an average word count of 251 words, with a Flesch-Kincaid grade level of 12.6, showing a difficult level of readability. Among the evaluated responses, 21 (81%) had a median score of 4 or above, indicating a high level of accuracy and reliability (Table II). The responses garnered a median score of 4, reflecting good/only minor nonharmful inaccuracies. Five responses received a median score of ≤3, indicating poor information and minor potentially harmful inaccuracies. The Fleiss multirater kappa value indicated moderate agreement (κ = 0.42, *P* < .001).^8^

**Table II:** Summary of rating of ChatGPT responses

Question	Median	Range	Interquartile range
1	5	4-5	5-5
2	5	4-5	5-5
3	4	4-5	4-4
4	5	4-5	5-5
5	4	4-5	4-4
6	3	3-5	3-3
7	4	3-4	4-4
8	5	3-5	5-5
9	2	2	2-2
10	4	3-4	4-4
11	4	3-4	4-4
12	5	5	5-5
13	4	4-5	4-4
14	3	3-5	3-3
15	4	4-5	4-4
16	4	3-4	4-4
17	4	4-5	4-4
18	5	4-5	5-5
19	4	4-5	4-4
20	3	1-4	3-3
21	4	3-4	4-4
22	5	4-5	5-5
23	4	3-4	4-4
24	3	3-4	3-3
25	4	3-4	4-4
26	5	4-5	5-5

Accuracy of 26 answers from ChatGPT was rated with Likert scale from 1 to 5, as follows: 1, very poor/unacceptable inaccuracies; 2, poor/minor potentially harmful inaccuracies; 3, moderate/potentially misinterpretable inaccuracies; 4, good/only minor nonharmful inaccuracies; and 5, very good/no inaccuracies.

Our evaluation indicates that ChatGPT is a reliable source of information on a wide range of asthma-related topics. Nonetheless, we observed that some responses lacked depth and precision. For instance, when asked “What is the most sensitive test of asthma?,” ChatGPT delivered an extensive response, confidently stating that spirometry is the most sensitive test for diagnosing asthma, but it failed to present other diagnostic tests. We then challenged the response given by the chatbot, which revised its previous assertion, stating, “I apologize for any confusion caused. While spirometry is an essential tool in the diagnosis and management of asthma, it is important to note that it is not the most sensitive test for asthma.” The revised response included a bronchial challenge test with methacholine or histamine, but it still failed to mention the mannitol challenge test, reversibility tests with β_2_-agonist, and inhaled or oral steroids. Similar findings have been observed in other diseases, where AI-generated responses often exhibit high levels of accuracy but sometimes lack the necessary depth and fail to provide comprehensive context or overlook less common diagnostic options.^6^^,^^9^

Although ChatGPT has potential as an informative platform for asthma-related information, limitations should be acknowledged. One notable limitation is ChatGPT’s inability to provide citations, hindering professional users from independently verifying the accuracy of the information provided. Additionally, the model lacks real-time updating capabilities based on evolving medical knowledge, raising concerns about the accuracy of time-sensitive information over time. Furthermore, the average Flesch-Kincaid grade score of 12.6 (ie, college-graduate reading level) could limit its effectiveness, particularly in countries with lower literacy rates or uneven access to health care. In such regions, individuals from lower socioeconomic backgrounds or remote areas often have reduced educational opportunities, potentially exacerbating the challenge of understanding complex medical advice. The high reading level might cause misunderstandings or misinterpretations, potentially affecting health outcomes.

This study underlines the potential of ChatGPT in offering valuable insights into asthma, which could significantly contribute to patient education and awareness. The model’s ability to provide accurate and understandable information could empower individuals to take proactive steps in managing their condition. The potential of AI tools like ChatGPT to aid in continuous medical education is illustrated by Goodman and colleagues’ demonstration of AI’s adaptability in learning and improving its responses through reevaluation.^9^ This can be particularly beneficial in asthma management, where ongoing updates in diagnostics and treatment guidelines can be seamlessly integrated into AI platforms, ensuring that health care providers and patients receive the most current information. Although it should not replace professional medical advice, it can significantly affect patient education and engagement. However, addressing the limitations identified in this study is crucial to ensure the continued accuracy and usefulness of AI-driven platforms in health care information dissemination. Integration with reputable medical databases and real-time updating mechanisms could enhance ChatGPT’s credibility as a trustworthy and up-to-date medical information source.

Our research aimed to encompass a wide spectrum of asthma-related topics. However, we acknowledge that there are still further aspects of asthma that could be explored in future studies. Future investigations should study the user-specific applicability of ChatGPT, examining whether it is more beneficial for different user groups like patients, non-MD health care personnel, medical students, or clinicians. This exploration is crucial for understanding how ChatGPT’s responses can be optimally tailored to various levels of medical knowledge and informational needs. Additionally, as the landscape of AI evolves with the emergence of new chatbots, it becomes important to assess whether these newer models surpass ChatGPT in accuracy and precision for providing medical information.

**Clinical implications**

Platforms like ChatGPT have potential in asthma education, enhancing patient knowledge and engagement. However, addressing accuracy and the information’s currency is vital for reliable integration into patient care.

## Disclosure statement

Disclosure of potential conflict of interest: H. Meteran reports receipt outside the present study of honoraria for lectures or advisory board meetings from GSK, Teva, Novartis, Sanofi-Aventis, Airsonett AB, and ALK-Abelló Nordic A/S; and research grants from ALK-Abelló A/S. S. F. Thomsen reports, outside the present study, acting as speaker and/or advisor for 10.13039/100004339Sanofi, 10.13039/100006483AbbVie, 10.13039/501100023331LEO Pharma, 10.13039/100004319Pfizer, Eli Lilly, Novartis, UCB Pharma, Almirall, Union Therapeutics, and Janssen Pharmaceuticals; and receiving research support from Sanofi, AbbVie, LEO Pharma, Novartis, UCB Pharma, and 10.13039/100008897Janssen Pharmaceuticals. The rest of the authors declare that they have no relevant conflicts of interest.

## Supplementary data

Supplementary Table E1
