# Supplementary material for: Evaluating the scientific reliability of ChatGPT as a source of information on asthma
Source: J Allergy Clin Immunol Glob. 2024 Aug 28;3(4):100330. doi: 10.1016/j.jacig.2024.100330 (PMC11426030; doi:10.1016/j.jacig.2024.100330)
Supplement: Supplementary Table E1 [file mmc1.docx]

Table E3: Summary of responses with a median score of ≤3 (moderate/potentially misinterpretable inaccuracies)

| 6. Are there more than one type of asthma? | Yes, there are different types of asthma based on various factors. Some common types of asthma include:  1. Allergic asthma: This type of asthma is triggered by exposure to allergens such as pollen, dust mites, pet dander, or certain foods.  2. Non-allergic asthma: In this type, asthma symptoms are not triggered by allergens but can be caused by factors like exercise, cold air, respiratory infections, or irritants.  3. Occupational asthma: This type of asthma is caused by exposure to substances or conditions in the workplace, such as chemicals, dust, gases, or fumes.  4. Exercise-induced asthma (EIA): Physical activity or exercise can trigger asthma symptoms in individuals with EIA.  5. Childhood-onset asthma: Asthma that begins during childhood and persists into adulthood is known as childhood-onset asthma. It can have various triggers and may change over time.  6. Adult-onset asthma: Asthma that develops in adulthood, often without a childhood history of the condition. It can be triggered by allergens, respiratory infections, irritants, or other factors.  7. Aspirin-exacerbated respiratory disease (AERD): This condition involves asthma symptoms being triggered by the use of nonsteroidal anti-inflammatory drugs (NSAIDs), particularly aspirin.  8. Corticosteroid-resistant asthma: Some individuals with asthma may have difficulty achieving control of their symptoms with standard asthma medications, including corticosteroids.  These are just a few examples of the different types of asthma. It's important to note that asthma can vary in severity, triggers, and response to treatment among individuals, and a healthcare professional can provide a more accurate diagnosis and appropriate management based on an individual's specific condition. |
| --- | --- |
| 9. What is the most sensitive test of asthma? | The most sensitive test for diagnosing asthma is a pulmonary function test called spirometry. Spirometry measures the amount of air a person can inhale and exhale, as well as the speed at which they can exhale. It provides objective measurements of lung function and can help determine if there is airflow limitation, which is a characteristic feature of asthma.  During spirometry, the individual breathes into a device called a spirometer. They are asked to take a deep breath and then exhale forcefully and as quickly as possible into the spirometer. The spirometer measures several parameters, including forced expiratory volume in one second (FEV1) and forced vital capacity (FVC).  FEV1 is the volume of air that can be forcibly exhaled in the first second of the breath, while FVC is the total volume of air exhaled forcefully after a full inhalation. In asthma, there is often a reduction in the FEV1 compared to the predicted value, indicating airflow limitation. Additionally, spirometry can show improvement in FEV1 after the administration of a bronchodilator medication, which is another characteristic finding in asthma.  Spirometry is valuable in assessing the severity of asthma, monitoring its progression over time, and evaluating the response to treatment. It is also useful in differentiating asthma from other respiratory conditions with similar symptoms. However, it's important to note that spirometry alone cannot establish a diagnosis of asthma. The test results should be considered in conjunction with the individual's medical history, symptoms, and other clinical assessments to make an accurate diagnosis. |
| 14. If an adult man only has few symptoms per month can he then use SABAs whenever he has symptoms? | While short-acting beta-agonists (SABAs) can provide immediate relief during asthma symptoms, they should not be used as the sole or primary treatment for individuals who experience symptoms only a few times per month. It is important to understand that asthma is a chronic condition characterized by underlying airway inflammation, even when symptoms are infrequent.  In cases where asthma symptoms occur infrequently, but the individual is still diagnosed with asthma, the preferred approach is to use long-term control medications, such as inhaled corticosteroids (ICS), to manage the underlying inflammation and prevent symptoms from occurring. These medications are taken regularly, even when symptoms are not present, to maintain control and reduce the risk of exacerbations.  Using SABAs as the main treatment for infrequent symptoms is not recommended because it does not address the underlying inflammation and may lead to inadequate asthma control and increased risk of exacerbations over time.  It is crucial for individuals with asthma, regardless of the frequency of symptoms, to work with a healthcare professional to develop an appropriate asthma management plan. This may include regular use of long-term control medications and having a rescue inhaler (SABA) available for quick relief as needed. The healthcare professional can assess the individual's asthma severity, determine the most suitable treatment plan, and provide guidance on medication use, symptom management, and when to seek medical assistance. |
| 20. Can you treat asthma with exercise? | Exercise can be a beneficial component of asthma management and can help improve overall fitness and lung function. While exercise alone cannot cure asthma, it can contribute to better asthma control and quality of life. Here's how exercise can be used in asthma treatment:  1. Improved lung function: Regular exercise can help strengthen the respiratory muscles and increase lung capacity, leading to improved lung function. This can result in better tolerance of physical activity and reduced breathlessness during exercise.  2. Enhanced fitness: Engaging in regular exercise can improve cardiovascular fitness and endurance, which can positively impact overall health and well-being. Improved fitness levels can also make individuals with asthma more resilient to asthma triggers and better equipped to handle physical exertion.  3. Weight management: Maintaining a healthy weight is important for asthma management, as excess weight can contribute to worsened asthma symptoms. Regular exercise, along with a balanced diet, can help manage weight and reduce the burden on the respiratory system.  4. Asthma control: Studies have shown that individuals who participate in regular exercise may experience better control of their asthma symptoms. Exercise can help reduce airway inflammation and increase the effectiveness of asthma medications, resulting in fewer symptoms and a decreased risk of asthma exacerbations.  5. Exercise-induced bronchoconstriction (EIB): Some individuals with asthma experience symptoms triggered by exercise, known as exercise-induced bronchoconstriction or exercise-induced asthma. With proper management, including appropriate warm-up exercises, pre-exercise medication use (if prescribed), and gradual intensity progression, most individuals can still engage in physical activity and minimize exercise-induced symptoms.  It's important to consult with a healthcare professional before starting or modifying an exercise program, especially if asthma symptoms are poorly controlled or exercise triggers symptoms. A personalized exercise plan that takes into account an individual's asthma severity, triggers, and current fitness level can be developed in collaboration with healthcare professionals or exercise specialists. They can provide guidance on appropriate exercises, warm-up routines, and strategies to manage exercise-induced symptoms. |
| 24. I have an acute worsening of my asthma symptoms. What should I do? | If you experience an acute worsening of asthma symptoms, it is important to take prompt action to manage the situation. Here are some steps to follow:  1. Take your rescue inhaler: Use your quick-relief or rescue inhaler (such as a bronchodilator) as prescribed. It typically contains medications like short-acting beta-agonists that help to quickly open up the airways and relieve symptoms. Follow the instructions provided by your healthcare professional for the proper use of your inhaler.  2. Stay calm and monitor your symptoms: While experiencing an asthma exacerbation can be distressing, try to stay calm and focused. Pay close attention to your symptoms and monitor their severity. If your symptoms worsen or do not improve after using your rescue inhaler, seek medical assistance immediately.  3. Follow your asthma action plan: If you have an asthma action plan, refer to it for guidance on managing worsening symptoms. The action plan is a personalized document created by your healthcare professional that outlines the steps to take based on the severity of your symptoms. It may provide specific instructions on medication use, when to seek medical help, and any additional measures to take.  4. Contact your healthcare professional: If your symptoms do not improve or if they worsen rapidly, contact your healthcare professional or seek urgent medical care. Explain your symptoms, their severity, and any actions you have taken. They can provide further guidance, adjust your treatment plan if necessary, or advise you on whether you should seek immediate medical attention.  5. Avoid triggers: Try to identify and avoid any triggers that may have contributed to the worsening of your symptoms. These triggers could include allergens, pollutants, smoke, cold air, or exercise. Taking steps to minimize exposure to triggers can help alleviate symptoms and prevent further exacerbations.  Remember, asthma exacerbations can be serious and potentially life-threatening, so it is crucial to take them seriously and seek medical assistance when needed. It is advisable to have regular follow-ups with your healthcare professional to monitor your asthma control, adjust your treatment plan as necessary, and ensure you have an up-to-date asthma action plan. |
